# Characterization and Evaluation of Hydrothermal Liquefaction Char from Alkali Lignin in Subcritical Temperatures

**DOI:** 10.3390/ma14113024

**Published:** 2021-06-02

**Authors:** Madhawa Jayathilake, Souman Rudra, Naureen Akhtar, Alfred Antony Christy

**Affiliations:** 1 Department of Engineering and Science, Faculty of Engineering Sciences, University of Agder, 4879 Grimstad, Norway; madhawa.jayathilake@uia.no (M.J.); naureen.akhtar@uia.no (N.A.); 2 Department of Natural Science, University of Agder, 4630 Kristiansand, Norway; alfred.christy@uia.no

**Keywords:** lignin, HTL char, FTIR, SEM, TGA, carbonization, pores

## Abstract

An evaluation of hydrothermal liquefaction (HTL) char is investigated in this work. Morphological studies, N_2_ adsorption behavior, FTIR analysis, thermal behavior, and elemental composition are studied. The HTL char yield showed an increase with higher operating temperatures. It increased from 11.02% to 33% when the temperature increased from 573 K to 623 K. At lower temperatures, the residence time showed an impact on the yield, while close to the critical point, residence time became less impactful. Elemental analysis showed that both higher operating temperatures and longer residence times increased the nitrogen content of the chars from 0.32% to 0.51%. FTIR analysis suggested the char became more aromatic with the higher temperatures. The aliphatic groups present diminished drastically with the increasing temperature. Residence time did not show a significant impact as much as the temperature when considering the functional group elimination. An increase in operating temperatures and residence times produced thermally stable chars. HTL char produced at the lowest operating temperature and showed both the highest surface area and pore volume. When temperature and residence time increase, more polyaromatic char is produced due to carbonization.

## 1. Introduction

Numerous research groups study hydrochar from the hydrothermal carbonization (HTC) process. The morphological surface and chemical characteristics of that char have been investigated profusely. Besides, comprehensive studies on char from the hydrothermal liquefaction (HTL) process are sparse. HTL produces comparably less char yield than HTC. Nevertheless, with a small yield, the char from HTL can be utilized for a valuable purpose. Limited studies have suggested that hydrothermal char can help produce porous carbon [1]. Besides, hydrochar-based porous carbon can be an effective material in agriculture [2]. Porous carbon produced from hydrochar can be used as carbon storage in fields. Further, hydrochar and biochar’s (pyrolysis char) diverse abilities in the nitrogen cycling process were studied recently [3]. Notably, activated char can be used as an adsorbent for organic pollutants [4].

Lignin makes itself a valuable resource, being the second most common earthbound biopolymer available and the most significant naturally occurring source of aromatic compounds [5]. As a significant by-product of the paper and pulp industry, lignin is mainly used by paper mills to fuel energy recovery [6]. 

Hydrochar from HTC has been investigated thoroughly in recent studies. Falco et al. showed the strikingly different behavior of different chars from HTC of cellulose and glucose, where cellulose-derived char illustrated properties close to pyrolysis char [7]. Leng et al. investigated the hydrochar behavior of sewage sludge liquefaction with a morphological study and a study on the oxygen-containing functional group in char. Although the surface area and pore volume were low, oxygen-containing functional groups were high in char [8]. After that, their studies found that the rice husk-derived biochar was effective on Malachite green (MG) removal from the aqueous phase [9]. An illustrative study by Zhu et al. showed a positive correlation between the elemental compositions and the porous carbon’s porosity.

Furthermore, the authors observed that lower maximum temperatures and retention times lead to high porosity porous carbon [1]. Wahyudiono et al. observed that the functional group distribution of char from lignin changes drastically during decomposition in near supercritical water [10]. The impact of increasing temperature on the repolymerization of decomposed products from lignin was unearthed by Pinkowska et al. [11]. Char produced by the hydrothermal carbonization of cellulose, xylose, and lignin were studied by Kang et al., where the chars’ carbon content was mainly investigated [12]. Moreover, many studies have been carried out to study the decomposition of lignin in organic solvents [13,14,15].

Despite the considerable work on the HTL of lignin, most of studies have only focused on the liquid phase from the output. Focusing on the char may give insight into the decomposition pathways and possible ways to utilize the char as a helpful precursor for other value-added products, such as soil stabilizers for agricultural purposes and porous carbon production as well as anode production. Subsequently, pyrolysis char and hydrochar from HTC are widely studied, and the behavior and evolution of pyrolysis char and HTC char are widely accessible [1,16,17]. Besides, due to the different chemistry in the process and the complex reactions and degradation mechanisms, HTL char could offer different properties than pyrolysis and HTC char. 

Char is a by-product of HTL, where in most of the studies, the bio-crude product is given importance [18]. The motivation to study char extensively is to investigate the possibility of using HTL char effectively. It could be either in porous carbon production, as a fertilizer for agricultural purposes, or using it as a carbon capture material. Nevertheless, this study focuses on evaluating HTL char properties and characteristics with different operating temperatures and residence times. This study investigates the morphological, surface, thermal, and chemical characteristics of lignin-derived HTL char. The functional groups present in char are studied with Fourier transform infrared spectroscopy (FTIR), where the structural and surface behavior is studied with scanning electron microscopy (SEM). The thermal behavior of char is studied with thermogravimetric (TGA) analysis, and the nitrogen adsorption/desorption method is used to determine the surface and pore distribution of char. The chars are produced with different HTL operating temperatures and residence times to study and understand the different char-derived attributes with varying process parameters. 

## 2. Materials and Methods

### 2.1. Material

Being obtained from Sigma Aldrich Co., Oslo, Norway, the lignin used in this study was alkali lignin with a low sulfonate content and an average molecular weight of 10 kg/mole. In total, 16 mL of slurry was fed into the reactor. The lignin and water weight ratio was 1:9, which was maintained for all feedstock samples. All the analysis instruments used in the study are mentioned in the Section 2.3 characterization.

### 2.2. Experimental Procedure

Experiments were carried out in a 24 mL tubular steel reactor from Graco High-Pressure Equipment Inc. (HiP, Erie, PA, USA). A dead volume of 8 mL was kept in all the experiments to hold space for produced gasses and expansions. The reactor was purged with N_2_ to check for leakages and emit the air after the reactor was sealed. A fluidized sandbath was used to heat the reactor, while an external shaking mechanism connected to a frequency controller was deployed to shake the reactor during the reactions. In this study, three temperature values and three residence times were used. The temperatures used were 573 K, 603 K, and 623 K, while the residence times used ranged 10 min, 15 min, and 20 min. Therefore, every temperature value was studied with three residence times. For the experiments’ consistency, every experiment was repeated five times, and average values were used for analysis and are recorded below in Table 1. According to Table 1, each char sample’s carbon content stayed very similar regardless of the operating temperature or the residence time.

**Figure 1:**
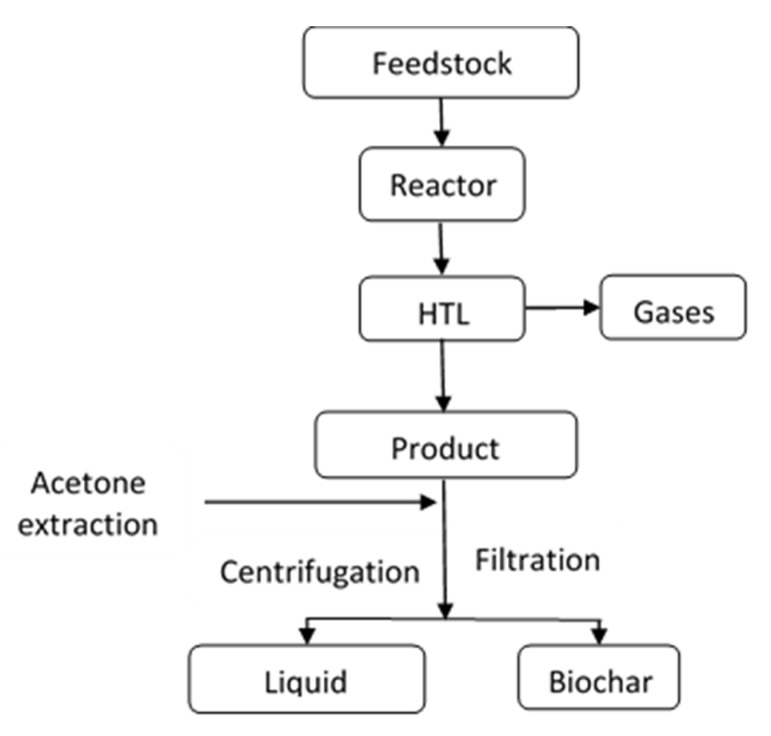
Char separation and extraction method.

After the desired residence time and the reactor were dismantled from the shaker and cooled with cold water for 30 min, acetone was used to extract the liquid and solid products from the reactor. The reactor was then washed with acetone four times after every experiment to ensure all the products were extracted. Furthermore, the solution was filtered, and the solid product was separated. To ensure that the solid and liquid phases were appropriately separated, solid residue (char phase) was again washed with acetone and filtered. Then, the char phase was weighted and dried at 378 K for 24 h before quantifying. A simple process diagram of the char separation and extraction from the HTL output is shown in Figure 1.

In this article, char yield denotes the amount of recovered solid residue after HTL, based on Equation (1) [19].

(1) $$Char\;yield\;\left(\%\right)=\frac{Char\;recoverd\;after\;the\;heat\;treatment\;\left(g\right)}{Initial\;amount\;of\;lignin\;used\;for\;the\;experiment\;\left(g\right)}\times 100\%$$

### 2.3. Characterization

Several different analytical tools and methods were used in this study to determine the char’s different properties and behaviors. 

According to ISO standard procedures, the lignin’s proximate analysis was performed using a muffle furnace LT 40/11/P330 (Nabertherm, Lilienthal, Germany). A PerkinElmer 2400 CHNS/O Series II elemental analyzer (PerkinElmer, Waltham, MA, USA) was used for the ultimate analysis. Oxygen as calculated by the difference of the other elements, where sulphur content was assumed to be negligible, although it is present in very low percentages in lignin. EN 15148 was applied to measure the volatile matter. In the elemental analysis, 1–1.5 mg of sample weight was used in all the test cases where samples were weighted in an Sn capsule and then placed in the elemental analyzer. Calorific tests were carried out using an IKA C6000 global standard-type bomb calorimeter (IKA®-Werke GmbH & Co. KG, Staufen, Germany) according to the DIN 51900–1 standard test for solid and liquid fuels. For the morphological study, scanning electron microscope (SEM) images of the samples were obtained by using a JSM-7200F scanning electron microscope (JEOL, Tokyo, Japan).

Thermogravimetric analysis of the feedstock and the char samples was carried out using a Mettler-Toledo Thermal Analyzer (TGA, Mettler-Toledo, Columbus, OH, USA). Mainly, the TGA and derivative thermogravimetry (DTG) graphs were used in this study to analyze the thermal behavior of char. The temperature was raised from 303 to 1273 K with a heating rate of 20 K/min with nitrogen as the purging gas with a constant flow rate of 25 mL/min. The mass loss in the sample was calculated using Equation (2).

(2) Mass loss (%)=Initial mass (%)−Final mass (%)

The sample’s infrared spectrum was measured using a Perkin Elmer FTIR spectrometer (Perkin-Elmer Ltd., Cambridge, UK) equipped with a nitrogen-cooled mercury-cadmium-telluride detector. Each dried sample dispersed in KBr as a 10% mixture was placed in a sample cup of a Perkin Elmer diffuse reflectance accessory, and the surface of the sample was carefully leveled flat. The spectrum of finely ground KBr was used as the background. The spectrum was measured in the range 4000–600 cm^−1^, and a total of 32 scans were made at a resolution of 4 cm^−1^. The resulting average reflectance spectrum was then transformed into the Kubelka–Munk format and saved as the final spectrum.

N_2_ adsorption isotherms of hydrochar were determined at 77 K (NovaTouch, Quantachrome, Boynton Beach, FL, USA). The Brunauer–Emmett–Teller (BET) model was used to calculate the surface area [20]. Pore volume was evaluated with the quenched solid density functional theory (QSDFT), using the calculation model for slits and cylindrical pores on the adsorption branch [21]. Before each measurement of surface area and porosity, samples were degassed at 423 K for 6 h. The total pore volume was defined from the amount of N_2_ adsorbed at a *p*/*p*_0_ value of 0.99.

## 3. Results and Discussion

### 3.1. Char Yield

At 573 K char, the yield showed 11.02% *w*/*w*_0_, 11.06% *w*/*w*_0_, and 12% *w*/*w*_0_ at 10 min, 15 min, and 20 min residence times, respectively. When the operating temperature was increased to 603 K, the char yield showed a rapid increase to 31% *w*/*w*_0_, followed by a rise to 33.05% *w*/*w*_0_ at 15 min residence time and then to 34% *w*/*w*_0_ at 20 min residence time. At 623 K for all the residence times, the char yield stayed around 33% *w*/*w*_0_. According to Table 1, with increasing temperatures and longer residence times, the nitrogen content of the chars increased. Therefore, for all three operating temperature values, the longest residence time reported the highest amount of nitrogen in the char. Similar behavior of nitrogen content has been observed with hydrochar produced by HTC process [1].

Further, an increase in operating temperature also increased the char’s nitrogen content, where it showed the complete opposite to pyrolysis char, which showed a reduction in nitrogen content with the increasing operating temperature [22]. Other research that carried out the hydrothermal degradation of lignin has observed the same trend: the lowest solid yield is obtained at the lowest operating temperature, and then the yield is increased with the increase of the operating temperature [23,24,25]. Besides, in some studies, the residence times used are relatively different from the current study. The relatively low yield at 573 K could be due to the promotion of hydrolysis and a decomposition reaction at lower temperatures than the repolymerization or rearrangement reactions. The relatively high yields at 603 K and 623 K can be attributed to the promotion of repolymerization and rearrangement reactions. Repolymerization reactions become prominent with the higher operating temperatures and longer residence times, leading to the higher char yield at higher temperatures [24,25]. With the increasing temperatures, free radicals from the broken ether bonds could pair with other carbon atoms and create more rigid bonds towards cleavage, which would create char-like structures [26]. Below, Table 2 shows the char yield obtained at different temperatures and residence times.

### 3.2. FTIR Analysis

**Figure 2:**
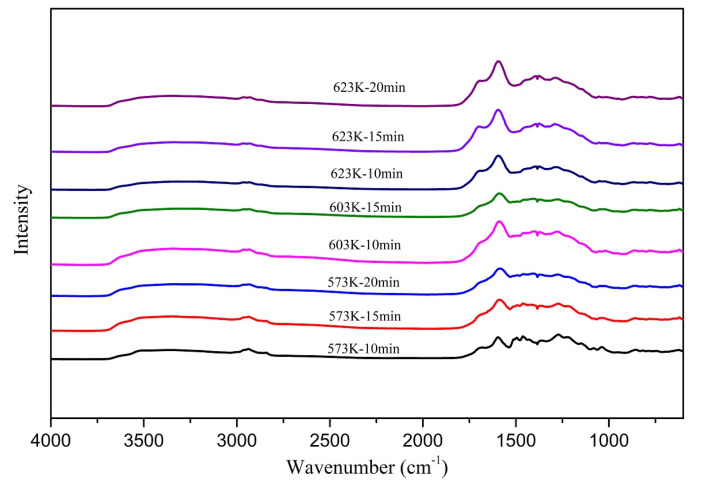
FTIR spectra of the chars.

The normalized FTIR spectra of the chars are shown in Figure 2. All the char samples obtained at different operating temperatures and residence times were analyzed, and the FTIR spectrums were obtained. In general, from the color and the spectra, it was apparent that the samples contain very little aliphatic content. The minor aliphatic character appearing in the low-temperature samples seemed to disappear at a high-temperature treatment. The aliphatic content also reduced in the samples treated at longer residence times.

**Figure 3:**
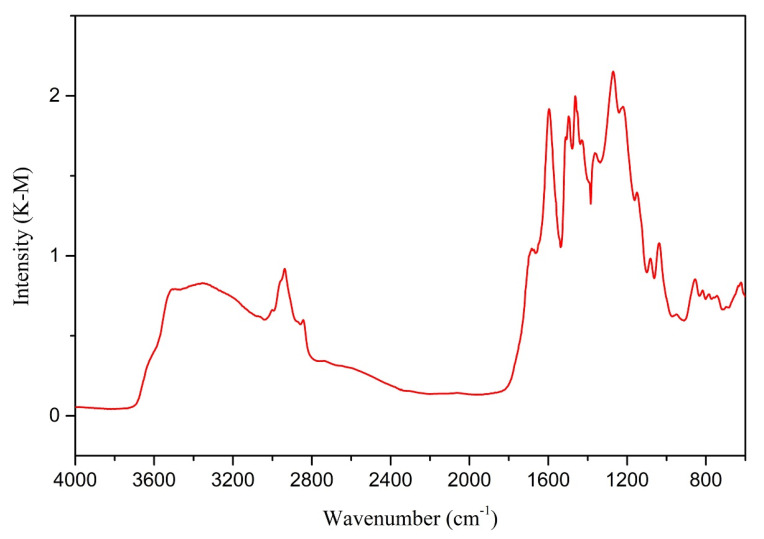
FTIR spectra and the available peaks of char from 573 K and 10 min residence time.

Figure 3 below illustrates a closer look at the FTIR spectrum from the char sample obtained at 573 K and 10 min residence time, produced at the lowest temperature and the shortest residence time among all the samples. The fingerprint in the region 2000–600 cm^−1^ mainly arises from the condensed products containing O, N, and sulfur compounds of lignin used in the experiments. The area under the region 3000–2850 cm^−1^ is an indication of the aliphatic content. 

**Figure 4:**
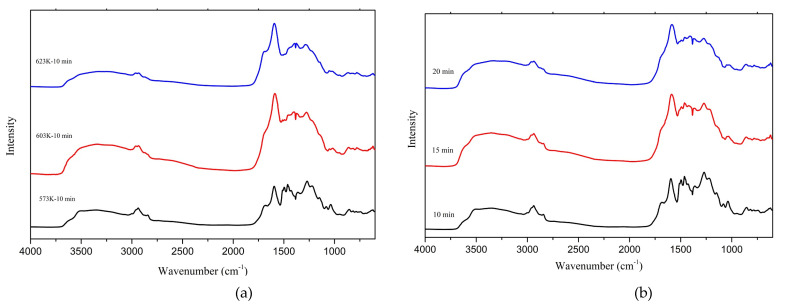
FTIR spectra of chars from (**a**) different temperatures with a 10 min residence time (**b**) different residence times at 573 K.

The FTIR spectra intensity variation reflects the variety of different functional groups as the temperature and residence time increase [27]. Figure 4a below shows the normalized FTIR spectra of the chars obtained with a residence time of 10 min at different temperatures of 573 K, 603 K, and 623 K, where Figure 4b shows the normalized FTIR spectra of the chars obtained at different residence times at 573 K.

The spectrum of char produced at 573 K with a 10 min residence time shows bands corresponding to aliphatic and aromatic -OH (3415 cm^−1^), aromatic ring modes (1595 cm^−1^ and 1140 cm^−1^), carbonyl group (1684 cm^−1^), aliphatic -CH_3_ (2944 cm^−1^) and CH_2_ groups (1453 cm^−1^), symmetric -CH_3_ stretching of the methoxyl groups (2851 cm^−1^), and symmetric -CH_3_ stretching (1035 cm^−1^) and C-H in Syringyl, Guaiacyl (857 cm^−1^ and 807 cm^−1^, respectively), as well as the nitro compounds (1353 cm^−1^) [28]. 

As temperature and residence time increase, the depth of aliphatic -CH_3_ (2944 cm^−1^), the symmetric -CH_3_ stretching of the methoxyl groups (2851 cm^−1^), -CH_3_ and CH_2_ groups (1453 cm^−1^), and the symmetric -CH_3_ stretching (1035 cm^−1^) [10,16], all decreased rapidly. This indicates how the polar functional groups decrease with increasing operating temperature and increasing residence time [29]. These groups are reduced to produce an aromatic char [10,16]. Such aromatic rings would result in producing fused aromatic rings after losing oxygen and hydrogen. Therefore, the increasing temperatures and the longer residence times promote fused ring production, resulting in more char. Although the aliphatic groups were eliminated from the chars produced at higher temperatures, many studies have shown that those aliphatic groups can be found in the liquid phase [11,24].

The peaks corresponding to Syringyl, Guaiacyl C-O (1272 cm^−1^) [30], which are characteristic of softwood lignin, started to diminish clearly. At 623 K and with longer residence times, most groups decreased. Subsequently, pyrolysis char and HTC char still presented some of the groups at 623 K [16,17]. Meanwhile, the small amount of aromatic and aliphatic OH (3415 cm^−1^) was seen to vanish with the increasing operating temperature. This can be mainly credited to the improvement of the dehydration reaction [31]. The band at 1595 cm^−1^ [10,16], corresponding to the aromatic ring and carbonyl group (1684 cm^−1^), seemed to be increasing drastically with the increase of operating temperature. This means the C-C and C-H bonds are consumed throughout the hydrothermal liquefaction process. Although the aromatic ring (1595 cm^−1^) increased with the longer residence time, it was not as significant as the operating temperature.

Meanwhile, the carbonyl group (1684 cm^−1^) seemed to decrease with the longer residence time. A possible reason for this observation could be reduced carbonyl group consumption, C-C, and C-H bonds with the longer residence time. Further, the nitro compounds (1353 cm^−1^) were observed to increase slightly with the increase of both the operating temperature and residence time. This can be attributed to the increase of nitrogen content in the char samples. 

O-containing functional groups in char can be used to measure using char as an adsorptive material. When the O-containing functional groups are enhanced, biochar has shown improved heavy metal sorption ability [32,33]. Although aromatic ring and carbonyl groups are increased with the increase of operating temperature, aromatic and aliphatic OH and Guaiacyl C-O are significantly decreased. Therefore, for particular purposes such as heavy metal sorption, optimum operating parameters should be used to obtain the maximum possible O-containing functional groups.

The longer residence times cannot help consuming C=C and C-H bonds effectively as the temperature does. Because of the dealkylation reaction, CH_3_ and CH_2_ groups are primarily removed from the chars, and this reaction could be more influenced by the operating temperature than the residence time. The fact that the aromatic ring keeps growing with the increasing operating temperatures and residence times means that an increase of fused aromatic rings is further observed. The chars’ aromatic nature increases with the rise in operating temperature monitored with pyrolysis char and hydrochar from hydrothermal carbonization [12,16,34].

The spectrum depicts a vanishing of the small aliphatic content with the residence time increase. Nevertheless, the reduction of the peak relating to the aliphatic content is slower with the residence time increase than the operating temperature increase. Therefore, both higher operating temperatures and longer residence times result in removing the aliphatic content from the chars in HTL. However, the impact of the operating temperature is more significant in removing the chars’ aliphatic groups than the residence time.

Monomeric radicals can be created by splitting weak bonds in the lower operating temperatures. The produced radicals can potentially create new radicals by attracting hydrogen to form monomeric phenolic compounds. When the operating temperature is further increased, C-C bonds can also be broken to create phenolic monomeric compounds. Meanwhile, these phenolic compounds can be polymerized and potentially produce more char as well. With the residence time increase, polymerization is further supported, and more char is made. When the operating temperature is further increased and comes close to the critical point, more fused aromatic rings could also be produced. In this study, the longest residence time used was 20 min. Besides, even at 623 K, increasing residence time did not significantly increase the char yield. Therefore, the impact of residence time on producing phenolic monomeric compounds and generating more char with polymerization must be studied further with longer residence times. Hydrothermal char could have certain advantages over pyrolysis char, such as easy decomposition, easy feedstock preparation, abundant functional group availability, and the possibility of coating pre-formed nanostructures with carbonaceous shells [35]. 

### 3.3. Thermal Stability of Char

**Figure 5:**
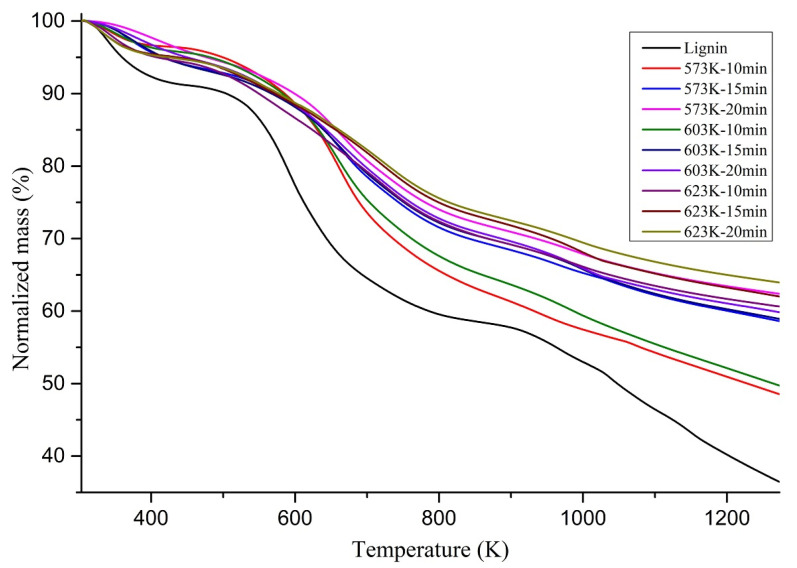
TG curves of lignin and the chars at 20 C/min heating rate under N_2_.

Char residues are studied for their thermal stability in an N_2_ atmosphere with a 20 K/min heating rate. Mass loss (TGA) curves of the lignin and chars are shown in Figure 5.

According to Figure 5, the chars from hydrothermal liquefaction are further heated and decomposed until temperatures reach up to 1273 K. A mass loss due to water vaporization can be observed around 373 K. Chars obtained at lower operating temperatures decomposed earlier. Furthermore, at the same operating temperature, chars produced from longer residence times took longer to decompose than the chars produced at shorter residence times, which should be attached to the remaining active functional groups. This can be supported by the FTIR results shown in Section 3.2, which clearly showed the decrease of the availability of aliphatic functional groups and the increase of aromatic functional groups in chars produced with increasing operating temperatures and increasing residence times.

**Figure 6:**
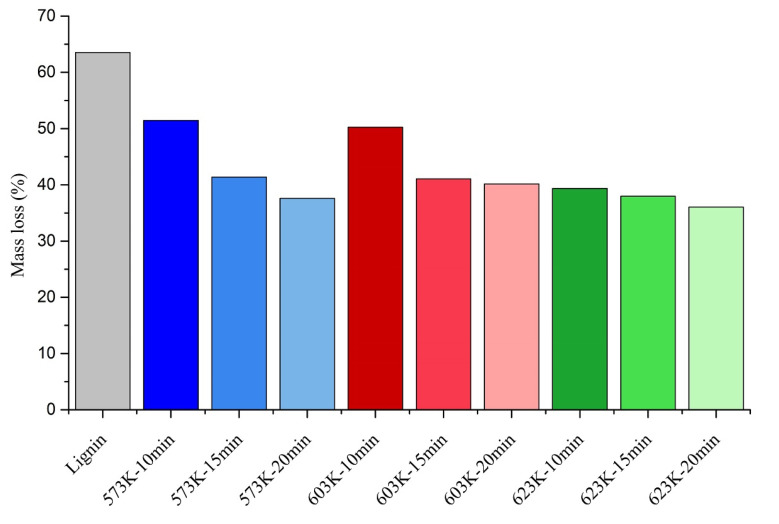
Mass loss of lignin and the HTL chars.

At 1273 K, the remaining solid residue percentage increased with the hydrothermal liquefaction operating temperature of chars, extending from 36.47% of the original lignin to 63.95% of the char prepared with 623 K and 20 min residence time. This observation indicated that more thermally stable structures are established at higher hydrothermal liquefaction operating temperatures and longer residence times. Similar behavior has been shown in pyrolysis char from lignin as well [36]. Below, Figure 6 shows the mass lost from each char sample at 1273 K as a percentage. It also presents a clear connection between the operating temperature, residence time, and the remaining solid residue. For each hydrothermal liquefaction operating temperature, the char produced with the shortest residence time showed the highest percentage mass loss. In contrast, the longest residence time showed the most negligible percentage mass loss. 

Chars produced with lower operating temperatures and shorter residence times could have more volatiles in them and weaker bonds in the chemicals in them. That could be a possible reason for the higher mass loss percentage. When FTIR analysis conclusions are taken into consideration, the chars become more aromatic with increasing temperature and residence time. Thus, the stability of the chars could go up with stronger bonds in the aromatic rings. Therefore, creating more stable structures with higher operating temperatures and longer residence times is a finding that can be drawn from this study.

**Figure 7:**
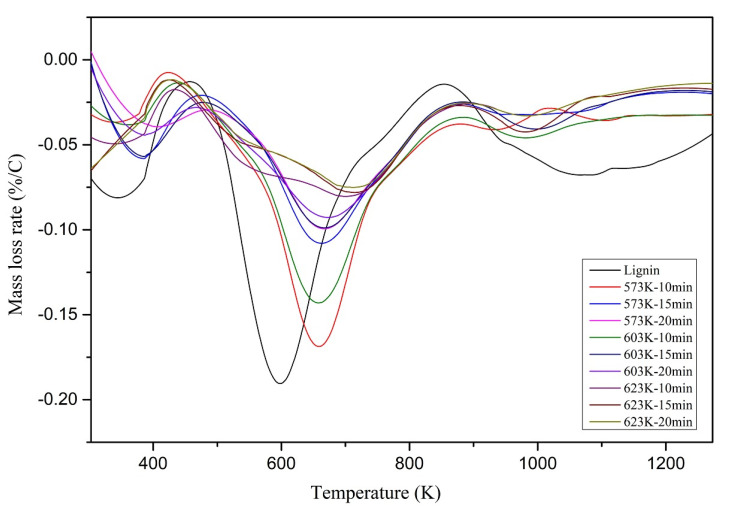
DTG curves of lignin and the chars at 20 C/min under N2.

Mass loss rate (DTG) curves of the lignin and chars are shown in Figure 7. According to the DTG graphs illustrated in Figure 7, the maximum mass loss peak was reduced and moved towards higher temperatures with the increasing HTL operating temperatures and residence time. This indicates that the chars produced at higher temperatures and residence times took longer to decompose and decomposed at higher temperatures due to more thermally stable structures.

The mass loss peak observed from 300 K to 420 K could be due to the moisture loss and the low boiling organic compounds loss. This is a widespread phenomenon with hydrochar. The maximum mass loss peak observed from 500 K to 800 K could be due to the carboxylation and cleavage of methoxyl groups. Moreover, the height of the mass loss peak was considerably reduced towards the higher HTL operating temperatures. This could explain the elimination of the methoxyl groups from the chars produced at higher HTL operating temperatures and longer residence times. Secondary reforming reactions of aromatic carbon skeletons could be the primary source for the evolution after 750 K.

Biochar interacts with soil fractions in different ways, and such interactions determine the influences on soil fractions by biochar [37]. Since the properties of biochar or HTL char are determined by the process conditions and feedstock properties, the influence on soil directly depends on how the char is produced. Biochar could positively and negatively impact soil, such as water-holding capacity, surface area, and bulk density [37]. Since HTL char and biochar chemistry are similar, these facts can also be actual with HTL char. Moreover, more stable char can be helpful from a climate mitigation point of view and regarding agronomic effects [38]. Further, with HTL char, higher operating temperatures and residence times produced more stable chars while also having higher nitrogen content. Therefore, chars produced at higher operating conditions and residence times could be beneficial in soil.

### 3.4. Adsorption of N_2_ at 77 K

The surface area, pore size distribution, and pore volume are the main factors that can explain the chars’ solid internal structure. The Brunauer–Emmett–Teller (BET) surface area and the density functional theory (DFT) pore volume of different chars obtained by hydrothermal liquefaction are shown in Table 3. For the N_2_ adsorption studies, chars produced at a residence time of 10 min at 573 K, 603 K, and 623 K temperatures are used.

**Figure 8:**
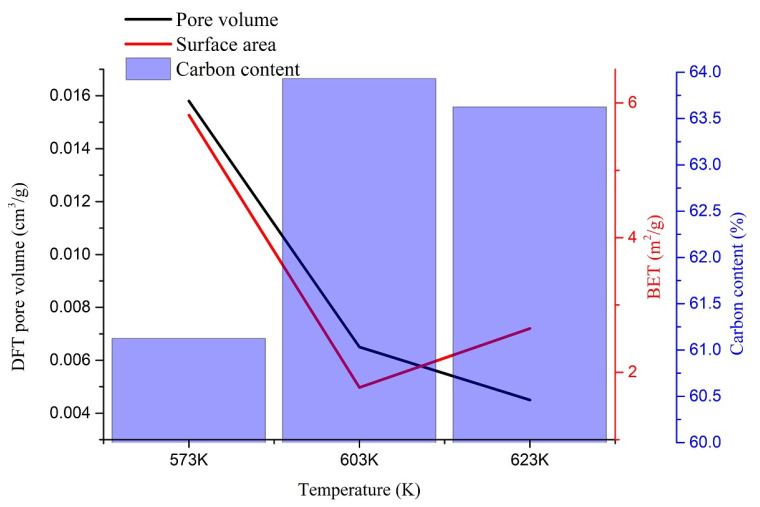
Relationship of carbon content surface area and the pore volume.

Below, Figure 8 shows the behavior of the Brunauer–Emmett–Teller surface areas and the quenched solid density functional theory (QSDFT) pore volume of different chars with their respective carbon contents in the chars. The lowest operating temperature produced the highest pore volume and surface area, where the pore volume shrunk with the increasing HTL operating temperature of the chars. The surface area showed the behavior of inverse dependability to the carbon content of the sample. Nevertheless, this behavior must be further investigated to observe a strong relationship between the surface area and the sample’s carbon content. 

The chars produced at 573 K showed both the highest surface area value and the highest pore volume value. The surface area of HTL char produced at 573 K (5.82 m^2^/g) was considerably higher than of the lignin pyrolysis char (about 0.5 m^2^/g) and pyrolysis char from wood (2.39 m^2^/g) produced at the same temperature [16,39]. At 603 K, both pyrolysis char (about 2 m^2^/g) and HTL char (1.77 m^2^/g) showed similar surface area values, whereas the HTL char produced at 623 K (2.65 m^2^/g) showed a lesser value than the pyrolysis char (about 5 m^2^/g) produced at the same temperature [16]. Nevertheless, the surface area of both HTL and pyrolysis-derived char ranged in the same values. The chars’ surface area exciting behavior reduced the surface area at 603 K and increased it at 623 K. 

**Figure 9:**
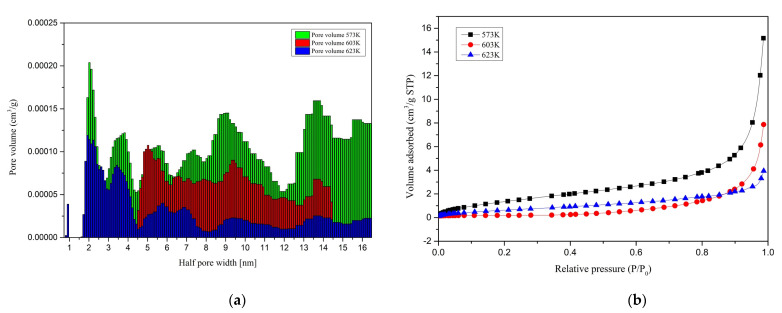
Adsorbed volume distribution (**a**) against micropore radius and (**b**) against relative pressure.

Pore volumes between 1.45 and 32.7 nm diameter were calculated by the QSDFT adsorption method. The maximum pore volume of 0.0158 cm^3^/g was observed with char produced at 573 K, whereas the lowest value of 0.0045 cm^3^/g was observed with the char produced at 623 K. Similar pore volume variation was observed with hydrochar produced by hydrothermal carbonization (HTC) [1]. Nevertheless, the pore volume of pyrolysis char from wood at 573 K was smaller (0.00256 cm^3^/g) than the values reported here [39]. Since the hydrochar (from HTC process)-based porous carbon inherits its parent material properties, the HTL char produced at 573 K may produce porous char with the highest porosity [1]. Nevertheless, to investigate the behavior of porous carbon from HTL, porous carbon produced from HTL must be further investigated with different activator kinds. The adsorbed volume distribution against micropore radius and adsorbed volume distribution against relative pressure for all three temperatures are shown in Figure 9a,b, respectively.

Chars obtained at 573 K and 623 K showed a wide range of pore diameter distribution from 1.45 nm to 32.7 nm, whereas the chars produced at 603 K were limited to a pore distribution from around 9 nm to 29 nm. The adsorption volume of chars produced at 573 K and 623 K were observed to be 2.04 × 10^−4^ to 1.01 × 10^−4^ cm^3^/(g·nm) and 1.19 × 10^−4^ to 6.74 × 10^−6^ cm^3^/(g·nm), respectively, whereas for char produced at 603 K, the adsorption volume was between 1.08 × 10^−4^ to 8.85 × 10^−6^ cm^3^/(g·nm). No pores over 30 nm were observed with the char produced at 603 K. Nonetheless, all the samples showed an incomplete pore distribution close to the most minor pore size limit. Although the pores’ distribution was a bit more complicated with the three different char samples, more conclusions could be drawn from the following section’s morphological analysis.

According to Figure 9b, in the low relative pressure region from 0.2 to 0.8, a slight increment in the adsorption volume was observed with all the samples indicating the mesopores’ existence with a broad distribution. Meanwhile, a sudden increase was noted in the relative pressure region from 0.8 to 1.0, showing a small microporosity of chars produced at all three temperatures [34,40]. Overall, char produced at 573 K showed a greater porosity.

In the HTL temperatures, volatiles are formed due to the strong decompositions. These volatiles could initiate the char structure’s pores when cooled if they are not scattered to the water medium. Higher temperatures might be capable of diffusing the volatiles faster. Therefore, the pores are not created abundantly. Moreover, the chars produced at 603 K had a higher carbon content and might have stacked better than the other chars produced at different temperatures. This could be a possible reason for the low surface area at 603 K. However, chars produced at 573 K showed the highest surface area, which can be attributed to the higher amount of volatile matter production during the liquefaction. This fact was ultimately proven by the char produced at 573 K having the highest pore volume among all three samples. At high temperatures, pores can be shrunk, resulting in macropores collapses [34]. Another explanation could be that due to further carbonization, pores could be melted, fused, combined, collapsed, or filled up by material around the pores [41]. This can be a possible reason behind the lower pore volumes of chars produced at higher temperatures. Due to the small pore sizes, CO_2_ adsorption is preferred over N_2_ adsorption for porosity and surface area analysis of HTC char. Therefore, the same basis could be applied to HTL char as well [35].

HTL char can be used for many applications, including HTL char-based catalysts, biochar-supported metal catalysts, and the availability of high surface functional groups as carbon storage to reduce fertilizers’ use in agricultural farms. Activated production for anode production for battery technology is also a possibility. Due to the high porosity, char produced at 573 K can be a good precursor for porous carbon production since it boasts considerably higher porosity to biochar produced at the same temperature.

### 3.5. SEM Analysis

**Figure 10:**
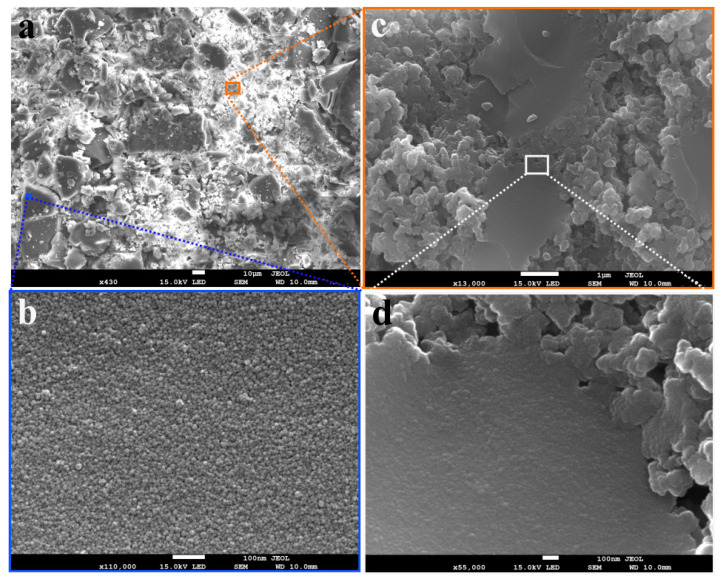
SEM image taken at low magnification for the char produced at 573 K and 10 min residence time; (**b**) high magnification (×110,000) image of the area indicated as blue color small square in part (**a**); (**c**) high magnification (×13,000) image of the area indicated as orange color small square in part (**a**); (**d**) high magnification (×55,000) image of the area indicated by a white color square in part (**c**).

Scanning electron microscope (SEM) images of the char sample obtained at 573 K are shown in Figure 10.

As shown in Figure 10a, a significant part of the char sample formed at 573 K consists of large particles (>10 um) with a smooth surface. A high-resolution SEM image showed the spores distributed all over the surface of these particles (Figure 10b). However, a small portion of the char sample showed smaller particles with a rough surface, where many vesicles adhered to the surface could be seen (Figure 10c). During char formation, lignin particles soften, melt, fuse, and release volatile materials [41]. The emission of volatile materials leads to the formation of open pores, consequently increasing the surface area. However, vesicles are often formed and adhere to the surface if volatiles are not completely diffused out [42]. This may result in pore blockage and, consequently, a reduced surface area.

At low operational temperatures (<603 K), the hydrothermal char is reported to exhibit minimum vesicle formation compared to the pyrolysis char, and this inhibition is suggested to be due to the permeation of water into the pores [42]. This is also consistent with the N_2_ adsorption studies presented in the previous section. A significantly higher surface area and pore volume were observed compared to the pyrolysis char.

**Figure 11:**
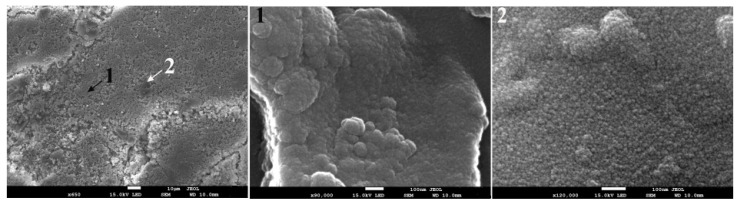
SEM images of the char produced at 603 K and 10 min residence time. High magnification images of the regions marked as 1 and 2 are displayed in the middle and the right panel respectively.

Hydrothermal char produced at 603 K showed most of the phase with small particles with a large number of vesicles adhered to the surface. A small number of larger particles with a relatively smooth surface was also present (Figure 11).

**Figure 12:**
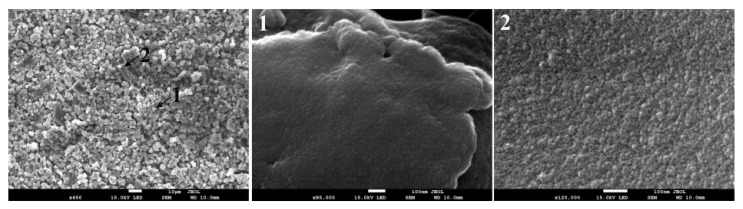
SEM images of the char produced at 623 K and 10 min residence time. High magnification images of the regions marked as 1 and 2 are displayed in the middle and the right panel respectively.

High-resolution images showed the presence of pores on the surface of these particles. An increase in vesicle formation with the rise in operation temperature could be due to the strengthening of the degradation process and intense release of gaseous materials that did not entirely diffuse out and subsequently condensed during the cooling process. A further increase of the temperature to 623 K assists the release of gaseous materials to some extent so that volatiles have sufficient energy to escape the lignin matrix timely [42]. This is evident from the SEM image in Figure 12 (left panel), where the larger particles with a smoother surface are observed to increase. These particles have minimal vesicles and a large density of open pores. This is consistent with the N_2_ adsorption results, where an increase in the char’s surface area was observed when the operation temperature increased from 603 K to 623 K.

### 3.6. Proposed Formation Pathway of Lignin Char at Hydrothermal Conditions

Lignin might not fully dissolve in water when the operating temperature is below 650 K [15]. Being a phenolic polymer, undissolved lignin can become polyaromatic char through solid–solid conversion [12]. Dissolved lignin can take part in hydrolysis reactions and produce phenolics in the process, which can be a main conversion route in this process. These phenolics can further polymerize and produce secondary char with aromatic properties. Additionally, polymerization can take place on the surface of the undissolved lignin as well. In low temperatures, lignin can be partially hydrolyzed because of the high bond energy.

Further, due to high ionic product and dielectric constant of water, ionic reactions are the most likely to happen. Mainly because of the hydrolysis reactions and possible free radical reactions, phenolic compounds can be produced [24,43]. In the FTIR analysis, the chars produced at 573 K showed relatively higher carbonyl, CH_3_, CH_2_, and OH groups. When the residence time and temperature are further increased, phenolic compounds such as guaiacol can be produced due to C-C bonds’ cleavage [24]. Nevertheless, guaiacol is an intermediate during the lignin liquefaction process and converts into catechol and phenols with increased temperature and residence time [6,11]. Furthermore, these phenolic compounds, such as guaiacol, can further polymerize and produce more char as well [24]. Around 573 K, guaiacol has shown the maximum production, and with the temperature increased, the production rate has gone down [24,43]. This could be a possible reason for the high surface area, the pore volume observed in N_2_ adsorption, and the chars’ SEM analysis. Besides, around 603 K, vesicles are often and abundantly formed because of the high degradation and fast and intense release of volatiles.

Nevertheless, the vesicles adhere to the surface when the volatiles are not completely diffused out. These closed vesicles create smooth surfaces. Thus, the surface area can be decreased at 603 K and then increased at 623 K since the pores are opened up due to the uncovering of vesicles.

**Figure 13:**
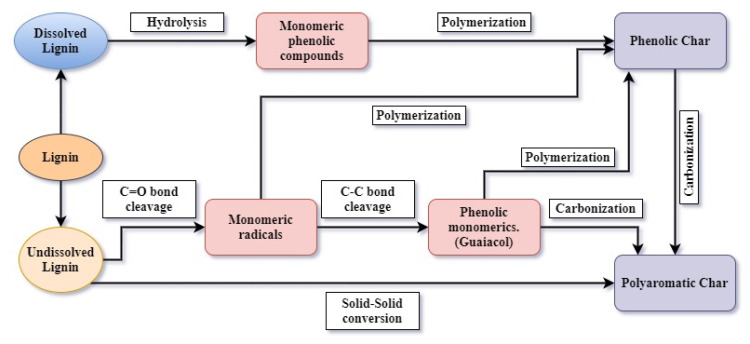
Reaction mechanism for char formation from lignin HTL.

The increase of peaks corresponding to the aromatic ring (1595 cm^−1^) and carbonyl (1684 cm^−1^) with the increasing temperature show the aromatization or the fusion of the chars’ ring structures. Further, the consumption of C-C and C-H bonds is also portrayed by increasing those peaks. When the temperature is around 603 K the CH_3_, and the CH_2_ groups start eliminating, the char has shown a more aromatic nature already. With the further incrementation of the temperature and residence time, fused ring structures can be formed by carbonizing the aromatic rings. Most of the functional groups except aromatic groups are expelled from the chars at this moment. Close to the critical point, radical reactions become dominant and more influential than ionic reactions due to the property change of water [24]. Because of the medium’s high radical nature, the phenolic radicals could depolymerize and contribute to the char phase. At this temperature, most of the volatiles may be emitted, and only a smaller amount of volatiles is available to leave the structure and create pores. This could be a possible reason for chars produced at 623 K showing the least pore volume and showing a significantly smaller surface area value than the chars produced at 573 K. Furthermore, the peaks corresponding to nitro compounds (1353 cm^−1^) show the increase of nitrogen compounds with the increase of the operating temperature and residence time. This could be mainly due to the aromatization process in the char [29]. With the results found and the literature data, a possible reaction mechanism for char formation from lignin HTL is illustrated in Figure 13.

## 4. Conclusions

HTL chars produced from lignin at 573 K, 603 K, and 623 K and residence times of 10 min, 15 min, and 20 min were studied. Both increasing operating temperature and residence time resulted in a positive impact on the nitrogen content of the chars.

The aromatic ring and the carbonyl group were strengthened drastically with increased operating temperature, and chars became more aromatic, while aliphatic groups were observed to vanish. Although the impact was considerably low, the same trend could be seen with the residence time too. Nevertheless, the carbonyl group showed contradictive behavior with temperature and residence time, where it showed an increase with increasing operating temperature, while a decrease was observed with the longer residence times.

With the increasing temperature, the pore volume decreased at all the operating temperatures, where the surface area showed the minimum at 603 K. According to the SEM analysis, the char sample formed at 573 K consists of large particles (>10 um) with a smooth surface. A high-resolution SEM image showed the presence of pores distributed all over the surface of these particles. Possibly because of the permeation of water into the pores at 603 K, minimum vesicle formation was exhibited by the char, compared to the pyrolysis char. Besides, at 623 K, the vesicles seemed to be opened and increased the surface area slightly.

Higher operating temperatures and longer residence times produced more thermally stable chars, where the residence time substantially impacted the thermal stability of chars produced at 573 K. At lower temperatures, the cleavage of the weak bonds can be seen, where polymerization was observed at higher temperatures and longer residence times. Carbonization is the main process of creating char. Longer residence times helped create more polyaromatic rings by assisting further carbonization.

## Figures and Tables

**Table 1:** Ultimate analysis results of the experimental samples (wt.% dry ash-free).

Sample	C	H	N	O
Lignin	51.500 ± 0.102	4.120 ± 0.021	0.350 ± 0.031	44.030 ± 0.154
Char 1 (573 K—10 min)	61.125 ± 0.315	4.780 ± 0.02	0.325 ± 0.075	33.770 ± 0.070
Char 2 (573 K—15 min)	63.105 ± 0.295	4.455 ± 0.045	0.346 ± 0.061	32.094 ± 0.279
Char 3 (573 K—20 min)	61.800 ± 0.160	4.140 ± 0.010	0.453 ± 0.024	33.607 ± 0.126
Char 4 (603 K—10 min)	63.930 ± 0.210	4.125 ± 0.025	0.368 ± 0.022	31.577 ± 0.163
Char 5 (603 K—15 min)	63.845 ± 0.005	3.875 ± 0.065	0.503 ± 0.015	31.777 ± 0.075
Char 6 (603 K—20 min)	63.710 ± 0.172	3.759 ± 0.032	0.517 ± 0.017	32.014 ± 0.221
Char 7 (623 K—10 min)	63.625 ± 0.105	3.770 ± 0.022	0.407 ± 0.013	32.198 ± 0.112
Char 8 (623 K—15 min)	58.920 ± 0.930	3.395 ± 0.015	0.461 ± 0.021	37.224 ± 0.966
Char 9 (623 K—20 min)	63.380 ± 0.165	3.590 ± 0.014	0.512 ± 0.018	32.518 ± 0.197

**Table 2:** Char yields at different operating temperatures and residence times.

Sample	Char 1	Char 2	Char 3	Char 4	Char 5	Char 6	Char 7	Char 8	Char 9
Yield (*w*/*w*_0_ %)	11.02 ± 0.3	11.06 ± 0.15	12.00 ± 0.21	31.00 ± 0.4	33.05 ± 0.23	34.00 ± 0.16	33.03 ± 0.11	33.03 ± 0.16	33.05 ± 0.22

**Table 3:** Surface area and pore volume variation at different operating temperatures at 10 min residence time.

Sample	Surface Area (m^2^/g)	DFT Pore Volume (cm^3^/g)
573 K—10 min	5.82	0.0158
603 K—10 min	1.77471	0.0065
623 K—10 min	2.65	0.0045

## Data Availability

Data available on request due to the privacy. The data presented in this study are available on request from the corresponding author. The data are not publicly available due to ongoing research.
